# Supplementary material for: The interactome of intact mitochondria by cross-linking mass spectrometry provides evidence for coexisting respiratory supercomplexes
Source: Mol Cell Proteomics. 2017 Dec 8;17(2):216–32. doi: 10.1074/mcp.RA117.000470 (PMC5795388; doi:10.1074/mcp.RA117.000470)
Supplement: Supplemental Data [file supp_RA117.000470_133922_0_supp_23978_fzffwf.zip › spectra_annotation/new_mito_protein_spectra_annotation/219-1-247-1-607-1.pdf]

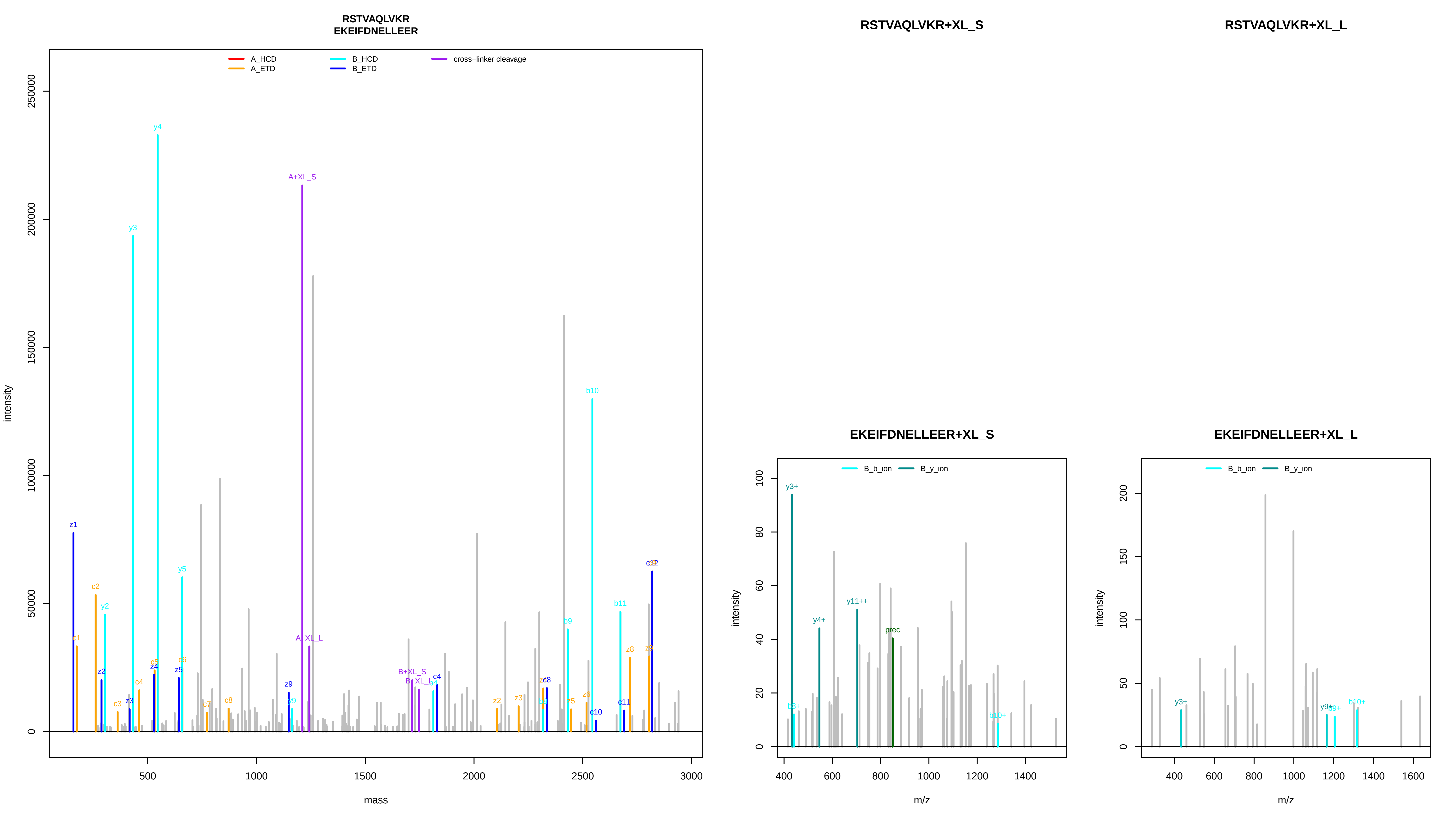
RSTVAQLVKR
EKEIFDNELLEER
A_HCD
A_ETD
B_HCD
B_ETD
cross−linker cleavage
intensity
mass
y4
y3
A+XL_S
A+XL_L
B+XL_S
B+XL_L
b10
b11
b9
y5
y2
z1
c2
c1
RSTVAQLVKR+XL_S
RSTVAQLVKR+XL_L
EKEIFDNELLEER+XL_S
EKEIFDNELLEER+XL_L
B_b_ion
B_y_ion
y3+
y4+
y11++
prec
b10+
y9+
m/z
